# Developing Authenticity, Building Connections: Exploring Research Methodologies in Asia

**DOI:** 10.1007/s11417-021-09358-z

**Published:** 2021-11-08

**Authors:** Sally Atkinson-Sheppard

**Affiliations:** School of Social Sciences, University of Westminster, London, UK

**Keywords:** Asian criminology, Research methods, Decolonising criminology, Ethnography

## Abstract

The article considers the methodological opportunities and challenges associated with three large-scale ethnographic studies conducted in Bangladesh, China, and Nepal. It reflects on how locally and regionally embedded cultural practises and meanings shape Asian criminological research projects. The article argues that conducting research in certain Asian contexts benefits from an awareness and sensitivity to specific modalities of culture in these regions. The following deliberations reflect on the importance of developing authenticity and building connections, embedded within concepts specific, and relevant to research in Asia—relationality, guanxi, patronage, and adda. The challenges of the research projects, of which there were many, are also discussed and include dichotomies between research conducted in the global North and global South, coloniality, ethics, and issues faced by a British researcher, conducting research in Asia.

## Introduction

In early 2021, I was asked to attend a conference to discuss how foreigners, like myself, are able to conduct research in Asia. My first thoughts were of ethics, reliability, and validity, the well-known and widely deliberated components of qualitative research (Englander, 2012; Silverman, 2010). I reflected on the years I have spent in Asia and the challenges I have overcome to conduct ethnographic research on sensitive topics—gangs, organised crime, and the exploitation of street children in criminal groups. However, while doing so I considered what really made my research work; beyond securing access, developing risk protocols, ensuring validity. It occurred to me that there are many factors marginalised in criminological research methods discourse; human values, hard to quantify, unsolidified but imperative, and meaningful. The values that facilitate access, the reasons why people spoke to me, often sharing incredibly personal and sensitive experiences, the process of building connections, contextualising data, exploring the nuances of research, and the diversity of participants.

This article summarises my thinking around the methodological opportunities and challenges I faced while conducting three large-scale ethnographic studies conducted in Bangladesh, China, and Nepal. I consider how locally and regionally embedded cultural practices and meanings shape Asian criminological research. I argue that conducting research in certain Asian contexts benefits from an awareness and sensitivity to specific modalities of culture in these regions. The following deliberations reflect on the importance of developing authenticity and building connections; arguing that researchers should remain reflective of the nuances of conducting research, embedded within concepts specific and relevant to research in Asia—relationality (Liu, 2017), guanxi, patronage, and adda. Nevertheless, some of the challenges of the research projects, of which there were many, are discussed here and include dichotomies between research conducted in the global North and South, coloniality, ethical issues, and the challenges I faced—as a British researcher, conducting research in Asia.

### Criminology and Research in Asia

The discipline of criminology and understandings of crime and violence are overwhelmingly based on studies into crime and justice within the global North (Carrington et al., 2016). This is embedded within wider social science discourse which seeks to ‘universalise’ theory, concepts and empirical research which remain largely untested outside of the ‘metropole’ (Connell, 2007). The focus, in many parts of the global North on ‘administrative criminology’, skews issues further; the reliance on (often) quantitative data fails to situate understanding within its wider social, political historical, and cultural context; in many instances ignoring the effects and legacies of empire (Blagg & Anthony, 2019) and coloniality (Maldonado-Torres, 2007).

Western centrism permeates research methodologies. Methodological textbooks focus on viability, reliability, and ethics but rarely consider how ontologies differ among researchers from the global North and global South (Cunneen & Rowe, 2014). Axiology which Cunneen and Rowe (2014 p56) argue, ‘refers to the set of values, ethics, and morality which underpin our research, including our ethical standpoint on the relationship of research to broader social or political goals’ remains underresearched, particularly within criminology. The effects of axiology on epistemologies, and the ways in which perceptions and ontology affect data collection and the validity of research remain marginalised in the discourse (ibid.). This leaves a great deal of scope for ‘re-centring’ discourse to prioritise axiology—with specific reference to authenticity and connection. The argument here is not to downplay the pragmatism of methodologies which seek to frame research fairly, accurately, and effectively but to generate a more expansive critique of research methods conducted outside of western contexts and which reflect (critically) on the positionality of global North knowledge and methodologies at ‘the centre’ (Carrington et al., 2016). My path as a British researcher incorporated as much ‘undoing’ as it did learning from extant ‘northern’ frameworks. On multiple occasions, the research discussed in this article demonstrated the limitations of global North theories^1^ and methodologies when applied in Bangladesh, China, and Nepal. This was particularly significant in regard to developing an awareness of specific modalities of culture and social connections in these regions, the nuances of which will be discussed as this article progresses.

The development of criminology in China is relatively recent, as Xu et al. (2013) explain, the ban on social science research was lifted in 1980 but the landscape of criminological research is often quantitative and focused on legislation and policy, rather than ethnographies from the periphery (ibid.). The landscape of conducting research in Mainland China is fraught with ethical and practical constraints, particularly for foreign researchers. As Wong (2002) explains, research involving collaboration between Chinese and Western scholars was, until relatively, recently prohibited. The ‘conservative political environment’ (Xu et al., 2013 p. 272), the challenges in obtaining public information (Wong 2002), the need to secure the support of authorities who are often suspicious of criminological research (Zhang et al., 2009) and threats of potential state action should research be considered to ‘reveal state secrets’ or ‘tarnish the reputation of the nation’ (Xu et al., 2013) mean that the landscape of criminological research in potentially risky for Chinese academics (Xu et al., 2013) and foreigners.

There are still relatively few studies which consider crime and violence in Bangladesh and Nepal and South Asian criminology has yet to develop a ‘distinct identity’ (Jaishankar, 2020: 353). Nevertheless, in Nepal, Sharma’s (2020) research discusses the criminal justice system, Subedi (2020) reflects on post-conflict crime and Sitoula (2020) explores victimological perspectives of human trafficking. In Bangladesh, Hossain’s research considers political crime victimisation, Nipun (2020) reflects on transitional justice processes, Khondaker (2020) explores victimisation among Rohingyas and Banjaree et al. (2020) study religious terrorism. All of which illustrates the rich diversity and complexity of crime across the region. However, there have been very few reflections on criminological research conducted by British researchers, in China or Bangladesh and Nepal. Furthermore, extant discourse provides limited guidance on how to navigate the specific forms of cultural exchange that dominate certain regions and societies in Asia. I propose that incorporating an awareness of locally embedded social connections, as well as developing reflexive authenticity helps to alleviate issues of positionality (i.e. coming from the global North) supporting the development of criminological research in Asia.

## Methodology

The reflections in this article draw three ethnographic case studies conducted in Bangladesh, China, and Nepal. The case study methodology is particularly beneficial as it allows for data to be collected over lengthy periods and from a variety of sources (Yin 2014). This methodology is advantageous for foreign researchers as to help to generate an understanding of context and enables and leads to greater validity of data due to opportunities for data triangulation (Bowling, 2010). The use of qualitative case studies within criminological research has illustrated reliability (i.e. Bowling, 2010) and has been utilised in Asia (see Wang, 2017); however, cross-comparative analysis of multiple research sites remains underdeveloped within the discipline.

The first study considered street children’s involvement in organised crime in Bangladesh and consisted of over 3 years of participant observation of the criminal justice system, 80 interviews (42 semi-structured and 38 unstructured interviews) with criminal justice practitioners, NGO workers and community members and a year-long embedded case study with a group of 22 street children, and the organisation that housed and supported them. I conducted this study while living and working in Bangladesh.

I also lived in China and while there initiated a collaborative research team involving both British and Chinese researchers. As a team, we conducted an extensive observation of Chinese society, a media analysis, and 99 qualitative interviews with criminal justice practitioners and community members from across China including Beijing, Shanghai, Hong Kong, Wuhan, Kunming, Xi’an, Shenzhen, Hubei, Shandong, and Tianjin. The participants included one ex-gang member and young people with friends or associates in gangs, 12 of whom were incarcerated in a young offenders’ institution in Shanghai, the majority for offences committed in groups.

I am currently conducting research in Nepal, alongside a collaborative research team; together, we have conducted a survey with 40 participants (the case study methodology differs from the previous studies due to COVID-19 restrictions) and 20 interviews with criminal justice practitioners, NGO workers and community members and which explored similar themes; namely the involvement of street children in gangs and organised crime. This project is underway, our aims are to continue research with adult practitioners and then, when COVID-19 restrictions allow, engage in an embedded case study with street children. This latter study differs as I do not live in Nepal but manage and conduct the research while living and working in the UK.

The research methods used within each study were specifically designed to allow for comparative analysis to occur. Each project consists of three main phases: an exploratory stage, interviews with criminal justice practitioners, NGO workers, community members etc., and research involving young people (to be completed, post COVID-19 restrictions in Nepal). The data sets from Bangladesh and China are largely similar—all 3 phases were conducted ‘face-to-face’. The Nepal study is slightly different, largely because of COVID-19 restrictions. For example, I have not been able to travel to Kathmandu to conduct the research and the research team and I are only able to carry out research online. This has a number of implications many of which are outside of the boundaries of this article to discuss however, our aim is to pursue ethnographic methodology, more in alliance with the previous studies when restrictions allow.

The analysis draws on a total sample set of 261 participants, across three countries and multiple research sites. There were convergences in the process: all participants received an information sheet and signed a consent form. Ethical approval was gained for each study.^2^ In addition, the article reflects on seven retrospective conversations (which explored motivations for engaging with the research and the research themes) with research colleagues, key collaborators and one participant, an adult human rights worker who lived on the streets as a child and has first-hand experience of gangs, organised crime, and the exploitation of children in criminal groups. Sharif^3^ played an imperative role in the Bangladesh study.

The similarity between the data collection methods aided the comparative analysis. Data were analysed in chronological order, with the Bangladesh study first. The data collected in China was analysed as a ‘stand-alone’ project, comparative analysis then occurred with the data set from Bangladesh. As with the previous studies, the Nepal data was analysed; comparative analysis between all datasets then occurred. The framework of convergences and divergences (Atkinson-Sheppard & Hayward, 2019) helped to frame the comparative analysis, the results of which are discussed as this article progresses.

## Discussion

### Developing Authenticity

The inception of any research project is couched within a wider notion of purpose; often wide-ranging and expansive, focused on research aims/hypotheses, the desire to generate information, contribute to knowledge. However, from my own experience, one of the ‘key research enablers’—developing a sense of authenticity, aligned with a desire to enact social change and make research ‘mean something’, is often discussed on the periphery. These implicit notions, likely components of many studies, are often marginalised in the discourse. This is not to say that research always affects change, the likelihood of which is ambiguous and difficult to quantify, nor that there are homogenous understandings of ‘meaning’ but rather to argue that centring motivations, aligned with challenging social injustice is beneficial, from an altruistic perspective but also for building connections, trust and rapport with participants, closely associated with authenticity. Furthermore, developing reflexive authenticity helps to develop culturally specific connections (discussed as this article progresses) and overcome issues related to positionality—in my case associated with my position as a British academic conducting research in Asia.

Authenticity is often embedded within social science discourse; its understanding is complex and multifaceted (Vannini & Franzese, 2008). Nevertheless, the imperative nature of ‘being authentic’ is a key component of many studies—particularly those that derive from an ethnographic perspective and prioritise an understanding of human behaviour, meaning and connection (ibid.). Vannini & Franzese, (2008) propose that authenticity is ‘being true to one self’ _being authentic is_ and is thus an emotional and self-reflective experience (p. 1). Developing an understanding of one’s own authenticity is a life-long process but an imperative component of developing reflexivity which, as many researchers argue is a significant component of qualitative research (Bryman & Cassell, 2006). It is also closely related to the purpose of a study—often personal and associated with a researcher’s individual motivations, interests and experience. Despite this, there are multiple challenges related to defining and developing authenticity. In many ways, I would have and still would benefit from more space to develop and reflect on my own authenticity, particularly the ways in which it evolves and relates to specific research projects, something which would also benefit from better integration into research methods paedology, specifically within the discipline of criminology.

The studies discussed in this article considered the involvement of street children in gangs and organised crime. The purpose was to consider a marginalised group who face stigmatisation, violence and oppression at the hands of organised crime groups, and in many instances the state (Atkinson-Sheppard, 2019). The axiology of the research was thus closely aligned with notions of ethics and morality, specifically related to upholding the rights of young people embroiled in organised crime. This purpose then influenced all other components of the research. The hypothesis of this article is that this axiology helped to generate a sense of authenticity—a sense that I, as the researcher believed in the purpose of the study—a belief greater than my own individual desire to complete a research study, achieve a PhD etc., and was able to generate interest and then build trust and rapport with my participants, many of whom shared similar motivations.

A retrospective conversation with one of my researchers illustrates her motivation for engaging with the study: ‘I wanted to understand their truth [young people], I wanted to have my input if we can help share their truth that’s how I feel I can contribute, how I can help. I feel like we share the same opinion on this the research you’re doing, your experience, we’re on the same page’. A research colleague based in Nepal elucidated further: ‘It’s not about the funding, the publications it’s how we can help, how we can prevent the street children from being harmed, exploited—that’s the purpose of our research’. One of the most important participants in my Bangladesh study Sharif, now an adult human rights worker but who had lived on the streets as a child described his motivations for engaging in the study. He explained how:

I was really interested in why the researcher wanted to learn about street children, this encouraged me to reflect back on the unexplored experiences of my life on the streets and my associations with gangs and the context in which it occurred. The War of Independence, losing my father, having to leave home to assist the survival of my family, arriving in Dhaka and living at a train station, learning to survive on the streets, making friends with other street children and becoming aware of criminal gangs, their involvement in crime and their motivations for doing so. I feel a real grievance that street children are largely ignored by society; they should be heard. The researcher felt the same way and gave me the opportunity to share my views in the hope that this may do something to share children’s experiences. I spoke to her on behalf of them, my friends the street children and the young people living on the streets, then and now.

It would be both naive and orientalist (Said, 1978) to propose that motivations for the research studies were entirely altruistic. A research colleague in China described how: ‘we both have similar interests and therefore lots to exchange but we also have different views, backgrounds and perspectives which can help to develop research’. A colleague in Nepal explained further:

Issues of globalisation help to frame the research in Nepal, we know the interlinks between crime types and between countries. Nepal is also closely associated with foreign countries—largely because of international aid the successful delivery of development and research projects which have a good reputation within the country. Foreigners are a welcome addition to research in my country. My own motivation for engaging in this research was structural and procedural—there are virtually no studies which consider a similar issue, there is a great deal of scope to explore this further and my own experience as an ex-police officer and researcher means I am very interested in the subject. Of course, the research attracts me on a personal level, I want to develop skills, expand my network but really, it is about wanting to help marginalised populations. I feel that we can do something to really help these street children.

As elucidated, and extensively discussed within research methodologies, the purpose of research is multi-faceted. However, ‘centring’ authenticity has wide-ranging and important benefits for criminological research. Framing research through the lens of authenticity helps to overcome issues of positionality, generate an ‘authentic environment’; a shared purpose and a sense of connection, the ability to generate a wider sense of purpose and meaning and situating the researcher in a position of reflexivity, moving away from individualist and egotistical motivations of achievement, success and ‘outcomes’ and into a shared space of understanding—and connection.

Authenticity changes and develops over time. The process of being an observing participant evolves, highlighting the importance of case study methodology (Yin 2014). In China and Bangladesh, I conducted years’ worth of participant observation, imperative due to my position as a foreign researcher, helping me to obtain an understanding of the social, cultural, historical and political contexts of the research. It was the part of the data collection that was often the most difficult to manage and analyse; page after page of personal reflections or field notes, hours spent reflecting on the nuances of issues, my position as a researcher and a multitude of other factors, challenging yet imperative to developing a sense of authenticity and understanding. In all research sites I relied on interpreters who became imperative parts of the research process. Temple (2002) argues there are two ways in which an interpreter can be used: ‘as a gatherer of facts or as an active producer of research’ (p 845). The studies utilised the latter approach. Interpreters were engaged in the research process, helped to secure access, translated the interviews, and were closely involved in the analysis (Temple, 1997). All of which greatly assisted my understanding of the nuances of language, ideas, and the culturally specific concepts discussed in this article as well as my ability to develop reflexive authenticity.

The Bangladesh study was my PhD and felt ‘authentic to me’ largely because I was responsible for the data collection and analysis. The year-long embedded case study with the street children provided the time and space to develop trust and rapport with the young people, ensuring the study was young person led and underpinned by strong ethical principles of child protection, confidentiality and anonymity (Atkinson-Sheppard, 2017a, 2017b). The more I learned about the realities of street children’s lives the more invested in the research I became (illustrated in a number of studies into street children, i.e. Hect 1998; Blanchet, 2008).

The China study was more collaborative, I worked alongside Chinese academics, a pattern replicated in Nepal. However, the study in Kathmandu has faced new challenges. The research team and I have re-designed our methodology to adhere to COVID-19 restrictions. Online interviews pose many questions for qualitative research, including how connections are sought and developed, authenticity is maintained. Defining an understanding of authenticity is complex but arguably, the ‘wider authentic’ nature of my studies increased the more I have engaged with researchers in each country; researchers who are authentic in their drivers for involvement, invested in making a change within their own countries and who provide a level of expert knowledge and access to participants that I could never emulate, no matter how long I lived within a country. This is concurrent with an ‘indigenous standpoint’ which argues that researchers should work closely with people and communities who truly understand the context in which the research occurs (Keane et al., 2017; Zavala, 2013), far beyond any understanding a foreigner can hope to achieve. The integration of an ‘authenticity lens’ within a researcher’s reflexive toolkit is thus nuanced, related to understanding one’s own authenticity, closely related to the positionality of the researcher, developing the sensitivity of the cultural context to the research, the locale, the timespan of the study, the association between the researcher, their participants, interpreters, and colleagues.

### Building Connections

Research, particularly of a qualitative nature relies on connection; connections between the researcher and participants, connections between data, and connections required for dissemination of research findings. However, the nature of developing and building social connections differs. This speaks to the dichotomy between the global North and global South in understanding connection—and proves to be an imperative part of the reflective process for global North scholars who engage in research in Asia.

As Liu (2017) argues, Asian societies are ‘relational’, this is in contrast to the individualism that permeates western societies. ‘Relationism culture’ leads to a research sphere which relies on social connections, this requires a paradigm shift from Western focused individualism that imbues social science research methodologies (ibid.). Relational cultural traditions affect relational personality traits which aggregate into relationism popular culture—this permeates life in Asia (ibid.). In contrast, individualist cultural traditions affect personality traits and thus research in the global North (ibid.).

However, as Liu (2017) explains, we are all a mix of individualism and relationism. Relationism is a pertinent factor in many western societies and in urban centres in Asia, individualism is over-taking the once predominant sphere of relational communities and embedded social connections. In ‘Crime and the Chinese Dream’ Bakken (2018: 2) describes China as ‘essentially a state capitalism dream with a patriotic and collectivist taint’ (p. 2), questioning the homogeneity of collectivism in Chinese society. There too are vast differences between notions of relationism between Asia countries, places, and communities (Liu, 2017). Relationism is embedded within complex processes of decolonization, recolonization, nation-building, class struggle, the reenforcing of racialized hierarchies, global governance, and consolidation of emerging elite power all of which have had profound impacts on the nature of culture in Asian societies (Ciocchini & Greener, 2021).

Nevertheless, from the perspective of my research and positionality as a white, British, female researcher, there were nuanced differences between conducting research in the UK and in Asia, many of which I associate with relationism. It is thus imperative to reflect upon the specific cultural nuances and social contexts of research. This article considers guanxi, a form of social capital often specifically associated with social connections in China (Zhang et al., 2009), patronage (closely associated with navigating social space in Asia), and adda (informal chats), of specific reference to the studies conducted in Bangladesh and Nepal. It is important to note that guanxi, patronage, and adda are not theoretical concepts in their own right, but terms used to explain social relations within the context in which they occur; this context is complex, vast and differs among the countries discussed in this article. Issues of power, particularly state power, coloniality, social structures, gender, age, and race all affect guanxi, patronage, and adda. Nevertheless, I utilised these concepts as a starting point and an important part of my ‘reflexive tool kit’ helping me to navigate research in Asia; often in light of the divergences between my experiences of conducting research in the global North, specifically the UK.

Xu et al., (2013: 274) argue that the collection of criminological data in China is constrained by the ‘distinct cultural context of negotiating access and collecting data’. This is closely related to guanxi: developing personal connections which support research facilitation (ibid.). The importance of guanxi has been discussed by a number of Chinese scholars. Wang (2017) explains how utilising existing family connections helped to facilitate access to participants in his research related to the Chinese mafia. Xu (2009) discusses developing ‘good guanxi’ as a research facilitation tool in his study of motorcycle taxi driver robberies. Wong (2019) describes the processes of developing connections which facilitated data collection for her study into the illegal wildlife trade. The importance of developing—and utilising existing guanxi is often fundamental to the success of a research study but is also, as Liang and Lu (2006) argue, time consuming and has particular relevance if the person holds a position of power within Chinese society. Guanxi is often considered synonymous with developing trust which Zhang et al. (2009) describe as an arduous but imperative component of securing access in China.

An associated concept is patronage. Lewis (2012) argues that ‘patron-client relationships are a cornerstone of society in Bengal, combining political, economic, and religious elements of social organisation’ (p. 156). Patron-client relationships operate in many spheres, within the job market, housing, education and social protection and provide context to relational interactions within Bangladeshi society (ibid.). Research considers the relationship between patronage and corruption (i.e. in China, Fun & Yao, 2018; Bangladesh, Lewis, 2012). However, patronage’s less nefarious impact remains under-explored in criminological research.

In all research sites, it was imperative to develop social connections or guanxi. I reflected on these concepts and my own, likely implicit bias towards individualism, in doing so I was able to forge new associations and support the research facilitation. This is not to say that the process was always easy or successful. At times, the prospect of developing guanxi, particularly in China was overwhelming. The extant literature is written from the perspective of Chinese scholars (i.e. Wang, 2017); there is very little information to guide foreign researchers in how to develop guanxi ‘in the field’. One of the ways I sought to tackle this was to utilise the contacts available to me via my own social network, largely consisting of journalists, Embassy workers and employees of International Government Organisations, and was as such biassed towards a ‘foreign lens’. Nevertheless, as the research progressed, I accessed NGO workers who supported migrant youth, taxi drivers, domestic workers, hairdressers etc., which provided new and important angles to the study. A 60-year-old migrant hairdresser, for example, became a key informant of the research and provided rich data in regard to migration and the wider context of children’s involvement in organised crime. It was only a year into the project that I met and began working with a collaborative research team, involving Chinese academics. In reality, my guanxi was loosely developed—and transient, reflective of my status as a foreigner in China. The importance of working alongside Chinese academics cannot be underestimated in this context; while I was able to develop some level of guanxi, it was my colleagues who were able to access police officers, prison officers and young offenders. It was obvious that my colleagues’ guanxi was more established and socially embedded in their own lives, families and locales—and thus more effective at generating access to ‘hard-to-reach’ participants.

In Bangladesh, my social connections felt stronger and more sustainable, supported initially by my employment at an International Development Organisation. This led to multiple contacts, some of whom developed into friends and participants. In many instances, these contacts had personal insights and experience of gangs and organised crime, reflective of the embedded nature of mastaan^4^ related behaviour in the country. They also had wide-ranging personal connections—and local knowledge. For example, I met Fardin^5^ shortly after arriving in Dhaka, he became a gatekeeper and one of the interpreters. Fardin’s expansive social network was an imperative part of the study, on his advice I met and interviewed multiple participants. I observed him utilising his patronage, often closely associated with his gender and patriarchy that imbues Bangladeshi society, of particular relevance when accessing male participants. At the same time, I was able to reflect upon and negotiate my own patronage in this context. The following describes Fardin’s help in greater depth: ‘Fardin enabled and facilitated my trips to slums and the backstreets of impoverished neighbourhoods; securing my safety and providing me with credibility. He was never afraid to correct me when I was wrong, pose questions to help develop my understanding or make me smile during the many hours we spent together in Dhaka traffic. Fardin introduced me to his family and friends. We celebrated Eid together; I met his children, grandchildren and visited the family’s village home. Whenever I return to Dhaka, Fardin is one of the first people that I call’ (Atkinson-Sheppard, 2019 p10)*.*

In addition, and with specific reference to Bangladesh and Nepal is ‘adda’ the notion, and importance of conducting ‘informal chats’ (Mahmud, 2019). For example, it would be ill-placed in Bangladesh to begin an interview without discussion of the participant’s family, their job and their views on political issues within the country. This ‘adda’ (informal chat/discussions) forms an imperative part of Bengali culture. It occurs on the streets, in neighbourhoods (referred to as parra addas), in the workplace, over cha (tea) within friendship groups, families, and is widely depicted in media and literature (Mahmud, 2019). Adda primarily consists of ‘chats’ about politics, although food, culture and family also play a role (ibid.). Imperatively, adda is an intrinsic part of Bangladeshi culture; it permeates and facilitates social relations and connections (ibid.). 

The opportunities of engaging in adda in research are multifaceted. First, it enables a connection to be made between the participant and researcher. Second, it develops opportunities for learning—in many instances adda led to discussions of issues I had not sufficiently considered, colonialism for example. Third, adda can lead to the development of wider social connections, in many instances participants recommended friends or colleagues for involvement in the research. Adda should thus play a part in any foreign researcher’s ‘toolkit’ for conducting research in South Asia, and likely wider afield. 

This is not to say that incorporating adda is straightforward, it requires an understanding of cultural context. For example, in Bangladesh, the political situation often forms part of any adda, at first; I struggled to engage in any meaningful political debate. Furthermore, it can, at times, be difficult to manage adda. On more than one occasion, I experienced issues of ‘research focus’ and timekeeping, related to a participant’s desire to discuss the context of the research rather than the research itself. Adda is often gender-specific, for example, I was able to engage in some conversations with women more easily than with men, likely illustrative of the patriarchy that exists in Bangladeshi society.

Adda and guanxi are closely related. Conversations were facilitated by existing connections where ‘common ground’ was easier to find. In addition, both adda and guanxi are affected by structures of power, particularly state power and the often-ambiguous role of law enforcers. For example, on one occasion Fardin’s ‘guanxi’ facilitated an interview with a Rapid Action Battalion (RAB) Officer. RAB officers in Bangladesh are involved in tackling some of the country’s most serious and organised crimes. They are also frequently accused of crossfire shootings and a wide variety of human rights violations (Uddin, 2018). The purpose of the interview, in my mind, was to discuss the landscape of organised crime. The RAB officer’s perception of purpose was very different. The informal adda at the start of the interview led to the RAB officer disclosing guilt and worry about his continued involvement with the RAB, he asked (with desperation) whether I could use my guanxi to help him transfer out of the RAB and find a new job. This was outside of the boundaries of the research, my social connections and was potentially unethical so I declined his request. We proceeded with the interview, but it was very challenging to manage both the officer’s expectations, display sensitivity in regard to his position as well as to continue with the data collection. The example demonstrates the potential merge into ambiguous territories for research and the often-complex dynamics related to guanxi, adda, power structures, and state crime.

Clearly, engaging in informal debate is a worldwide social phenomenon but there is something distinct and nuanced about adda in Bangladesh, learning, reflecting and then utilising adda as part of research engagement greatly benefited my study in Dhaka and formed an imperative part of my ‘reflexive toolkit’. However, developing social connections were reinforced and facilitated by the concepts discussed earlier, purpose and authenticity. A field note was written while in Bangladesh explains further:

When I reflect on the depth of my studies, the parts that it is based on purpose, it is when I made a genuine connection with a participant—a human connection, of knowing, empathy, and oneness. It is when I saw into the lives of street children through their own eyes when I visited people’s homes and celebrated their children’s birthdays, it is where the real connection and where ethnographic methodologies were personified.

### Research Challenges, Constraints, and Limitations

#### Positionality and Coloniality

In 1990, Quijano introduced the concept of coloniality/decoloniality, drawing on this work and early scholarship of Mignolo (1995), Maldonaro-Torres (2007: 243/244) distinguishes between colonialism and coloniality:

Coloniality is different from colonialism. Colonialism denotes a political and economic relation in which the sovereignty of a nation or a people rests on the power of another nation, which makes such nation an empire. Coloniality, instead, refers to long-standing patterns of power that emerged as a result of colonialism, but that define culture, labour, intersubjective relations, and knowledge production well beyond the strict limits of colonial administrations. Thus, coloniality survives colonialism.

As Ciocchini and Greener (2021) argue, neocolonialism is ubiquitous and deeply embedded within global structures of power, inequality, criminalisation, and repression including criminal justice systems and criminology. In many ways, criminology reinforces coloniality (Agozino, 2003). This is damaging to equality and fair and just criminal justice processes; it affects challenges to social injustice, is not reflective of most of the world today (Carrington et al., 2016), and unfairly advantages to researchers from the global North because of issues related to the production and dissemination of knowledge from the metropole, largely in the English language. As Mignolo and Walsh (2019) argue, we must tackle the ‘coloniality of knowledge’ and question the universal legitimacy of criminological research methodologies.

Coloniality affects and will continue to affect many parts of the global South. A participant in Bangladesh explained further:

There is a foreign mentality in Bangladesh, we were under British rule from 1757–1947, and the impact of this should not be ignored. Slavery is in our blood, not slavery like you might know it but slavery, where one person has to obey another, has to work for another. The British administration gives structure to our society—the hierarchy that existed then still impacts upon Bangladesh now. It is not just related to foreigners, if a senior person is in a room, people will stand up, salute. Colonial rule is still in place in our police departments, our prison systems, and it is linked to development. Opposition parties will always contact developed countries and try to get them to put pressure on the current government about human rights for example. After independence in 1971, a lot of money was pumped into Bangladesh and was not used properly. We are dependent on international aid and debt. Most money is retuned in international development in trade and consultancy with other countries. This has helped but also harmed Bangladesh in some ways, arsenic, and microcredit—scandal makes people poorer and poorer, life does not change, it is a trap of debt.^6^

These discussions highlight coloniality (Maldonado-Torres, 2007), the ways in which colonialism affects Bangladesh, the ‘patterns of power’ that frame politics, aid, development and culture (ibid.), and illustrate how ‘colonisation and the postcolonial are not simply historical events: rather, they are continuing social, political, economic, and cultural processes’ (Cunneen, 2011: 342). It also opens up discussion about how coloniality affects research and encourages us to ‘identify or interrogate the contemporary nature of current structures of colonialism’ (Ciocchini & Greener, 2021: 3).

Coloniality formed an embedded context to all of my research studies, illustrative of ‘existing imperialisms’ (ibid.:4). The fact that I am writing this article from the perspective of a global North scholar conducting research in the global South in itself reinforces coloniality, running ‘the risk of reproducing colonial epistemology and being unable to disentangle [discourse] from the hegemony of Western modern thought’ (Dimou, 2021: 1). As Dimou (2021: 1) argues, ‘our contemporary ways of being, interacting, knowing, perceiving, sensing and understanding are fundamentally shaped by coloniality’. The reality is that I was, and still am, ill-equipped to tackle these issues. The effects of colonialism and its impact on research methodologies did not feature as part of my post-graduate training, nor were they covered in any methodological paedology at the time and, while this is rapidly changing, there are lacunas in both teaching and practise, illustrative of the Western centrism that permeates criminological and wider social sciences (Carrington et al., 2016).

The most useful lessons on coloniality arose from my research participants. I remember many afternoons, as the sunset over Dhaka, where my colleagues and I would discuss Bangladesh’s history. These discussions acutely highlighted my lack of knowledge of the British Empire and its impact on the sub-continent and the contemporary coloniality that affects the region today. The challenges to reflect on coloniality are complex and multi-faceted, some of which are likely abated by better and more expansive inclusion of decolonising methodologies in research pedagogy but others which have to be learned in ‘the field’ and via personal commitments to understanding this imperative and complex subject.

Furthermore, the likelihood is that researchers like myself, have benefited from the coloniality that permeates global discourse and wider social sciences. This is juxtaposed with the challenges of being ‘outsiders’ within Asia. Nevertheless, these privileges are multifaceted, nuanced, and range from the production of knowledge to the overreliance on English as the mechanism by which research is disseminated and shared. These discussions of White—or global North ‘research privilege’ are not reflected sufficiently within Eurocentric criminological discourse or methodologies. Western academics conducting research and teaching methodologies run the risk of reinforcing coloniality via the neoliberal university, in the global North and beyond (Connell, 2007).

#### Ethical Reflections, Constraints, and Limitations

There are dichotomies between research which receives ethical approval in the West—and specifically in my case, the UK, but which is conducted in Asia. The research studies discussed in this article highlight how extant ethical procedures are insufficiently developed to support ethnographic research in Asia. For example, the Bangladesh study included an embedded year-long case study with 22 street children. The vulnerability of these children meant that gaining ethical approval for this study was (rightly) lengthy and intensive. However, the existing assumptions that permeate UK ethical guidelines fail to sufficiently reflect the realities of these children’s lives. This was evident in 2 main areas. First, as required by UK ethical procedures, should a participant disclose sensitive information that may pose a risk to themselves or others, I would have been under obligation to pass this information on to relevant services, possibly Social Services, or the police. There are, however, various issues that arise when attempting to apply this procedure in Bangladesh. Referring a street child to the police would likely put that child at increased risk of harm, not protection. This is associated with wide-ranging extant literature which considers police violence against vulnerable populations, including street children (Khatun & Jamil, 2013). A ‘child protection referral’ is unlikely in this instance due to the lack of Social Workers in the country (UNICEF, 2012). These issues, only apparent to me after living in Bangladesh for some time, posed a significant hurdle in assuring the safety of my participants while adhering to ethic procedures developed within the context of the global North and thus insufficiently nuanced to tackle these concerns in the global South. To address this, I developed a ‘Child Protection Policy’ specific to the study in Bangladesh (and relevant to the study in Nepal) which stated that, should a disclosure occur, or if I felt the child were ‘at risk’ or a risk to themselves or others, I would discuss the issue with the Director of the NGO, which supported the children at the time of the research, and we would work together to support the young person and others involved. This is not ideal nor viable for all studies facing similar issues, but an important tangible step in progressing the research and upholding the rights and safety of the participants. An additional component included the development of carefully considered interview questions, written to avoid potential disclosure of criminal offences, all of which was explained to the young people before the commencement of the study. The Child Protection Policy was not enacted in Bangladesh. It continues to inform the study in Nepal.

It is rare for children and young people to be involved in studies that consider an organised crime. This relates to ‘postcolonial paternalism’ and the ‘the dominant understanding of childhood in Europe which is closely interwoven with the process of colonisation’ Liebel (2019:1). ‘Western narratives of modernisation’ (Morrison, 2012: 3) denotes ‘children out of place’ with the dominant ideology of what a child should be and do; associated with innocence, education and play (ibid.). Yet these assumptions are ‘largely based on children, and childhood in the global North’ (Liebel, 2020: 2), leaving little room for discourse that represents children whose lives are not closely aligned within the dominant depiction (ibid.). This has implications for ethical procedures. There is an implicit assumption that children and young people should not know about the largely adult pursuit of organised crime. The research discussed in this article challenges this assumption. For many of the young people discussed in these studies, navigating organised crime was an imperative part of ensuring their survival on the streets. Questions are thus posed as to how ethical procedures can better reflect the realities of these children’s lives, protect their rights as participants but also celebrate their agency and engage young people’s voices in this challenging research terrain.

There are two final issues which impacted the research discussed in this article. Extant, global North ethical frameworks related to consent often, and understandably, require written consent. As scholars have argued, this is often difficult to obtain in Asian contexts. Wang (2017), drawing on his study into the Chinese mafia, describes how securing written consent and recording interviews is often a barrier to participant engagement. This was the case in China, but also in Bangladesh and Nepal (although recording interviews was possible in these contexts). Flexibility should thus be incorporated into ethical procedures to reflect the reality of obtaining consent and recording interviews in different contexts. A final dichotomy between research conducted in the global North and global South which has specific relevance to China relates to surveillance. It is widely acknowledged that foreigners in China may face some level of surveillance—in person or online. This is situated within a wider context of a risky and potentially hostile research environment, discussed earlier (Xu et al., 2013). Reports of censorship of research/the media are commonplace. It is outside of the boundaries of this article to consider these issues in any depth, and the reality is that I was and still am unable to fully comprehend the reality of this situation or any potential solutions. There is very little guidance available via existing global North frameworks to address this topic and from a Chinese perspective, there is (understandably) little written about the subject. However, the study in China illustrated the complexity of this issue, the importance of reflecting on surveillance from data protection, researcher and participant safety perspective, and the realities of conducting research in Mainland China today.

### Reflections on Divergence

It is important to consider the wider context of these discussions and reflect on the divergences between the research sites and locales. It is outside of the boundaries of this article to consider these divergences in any depth. However, the cultural, historical, and political contexts of all countries are vastly different. In Bangladesh, the weak state has been widely associated with political violence and instability (Moniruzzaman, 2009). In China, the widespread authoritarian nature of the Chinese state provides a vastly different context to gangs and organised crime (Wang 2019). Nevertheless, all countries have experienced significant and recent social change. Mao’s era and the Cultural Revolution ended in the 1970s at a similar time, Bangladesh gained its independence from Pakistan. The armed conflict of 1996–2006 saw many parts of Nepal controlled by ‘irregular armed groups, political parties, local armed groups, and criminal gangs but less by the state government’ (Thapa, 2019: 1), setting the ‘scene’ for understanding gangs and organised crime today.

The data in China illustrated significant differences between places. In Beijing, the streets are highly controlled meaning that the association of gangs and the streets, widely evident within extant global North literature is not necessarily relevant in this context (Atkinson-Sheppard ad Hayward 2019). In Hong Kong, the data demonstrated the existence of the Triads as well as South Asian gangs. In Shanghai, young people work as the ‘assistants’ of organised crime groups. In Tianjin migrant youth forms peer groups and Kunming and Southern China saw the existence of greater levels of sex trafficking and prostitution. In Bangladesh, the data was more localised and focused on Dhaka, but here too divergences occurred. The nature of mastaan groups differed, as did the slums that they controlled and the children that became embroiled in the lowest echelons of these criminal enterprises.

Migration formed an implicit part of all studies but varied; the mass migration seen in China was less evident in Bangladesh and Nepal but remained an explanatory factor for why young people become ‘streety-involved’. In Bangladesh, millions of children live on the streets, including in Dhaka the locale of the research (UNICEF, 2012). In Nepal, the numbers of street children are much lower but still present a concerning social issue. In China, millions of young people are involved in migration trajectories, causing some to become ‘street-involved’ and vulnerable to the advances of gangs and organised crime (Atkinson-Sheppard & Hayward, 2019).

In addition, and in regard to colonialism, Bangladesh was colonised by the British and then only gained its independence from Pakistan in 1971 (Jaishankar, 2020). Nepal’s locale closely related to India led to ‘colonised influences’ permeating the region despite the fact that it was never part of British rule (Gurung, 2018). China was not colonised by the British in the same way as neighbouring India and Bangladesh however, the effects of colonisation especially related to Hong Kong illustrates a more complex picture (Chan, 1996); the wider issues of western centrism in academia remain. These divergences make for a challenging research terrain, particularly in regard to comparative analysis.

The scope of this article is one of its biggest limitations. It was outside of the boundaries of this article to consider the depth of each research study, its associated methodologies and the complex undertaking of comparative research. This means that in many ways the article provides a somewhat superficial overview of three large-scale studies.^7^ It is important to reflect on my own understanding of the concepts discussed here. My own biases and my own positionality will have affected every part of these studies, some of which I have reflected on and some which require greater reflexivity and thought. However, despite these limitations, the article hopes to initiate conversations about the underpinnings of research conducted by foreigners in Asia in the hope that we can all learn or ‘unlearn’ more.

There are wider divergences. The work developed by Liu (2017) is illustrative of Asian criminology that, in reality, is mainly focused on research in East Asia (Moosavi, 2019). The emergent ‘southernizing discourse’ (Carrington et al., 2018) aims to reflect crime and justice in the global South but features a large number of Australian scholars (Jaishankar, 2020). As Jaishankar (2020: 354) notes, the relationship between the criminologies is fractured, leaving South Asian criminology facing ‘academic parochialism’, questioning the inclusivity, and connection between the discourses, within the context of western centrism discussed throughout this article.

## Conclusion

The discussions in this article reflected on the methodological opportunities and challenges associated with three large-scale ethnographic studies conducted in Bangladesh, China and Nepal. The article considered how locally, and regionally embedded cultural practises and meanings shape Asian criminological research*.* By considering relationality, guanxi, adda, and patronage I demonstrated the importance of developing awareness and sensitivity of specific modalities of culture in these regions. Developing authentic reflexivity in these areas helps to overcome issues of positionality, specifically related to global North scholars conducting research in Asia.

The challenges of the research projects, of which there were many, were also discussed and included dichotomies between research conducted in the global North and global South, coloniality, ethics, and issues faced by a British researcher, conducting research in Asia. These challenges highlight the difficulties of conducting research in Asia but also the opportunities. Most imperatively they demonstrate ways in which the discipline of criminology needs to develop and expand to better reflect research in the global South and challenge the historical legacies of empire as well as the omnipresence of coloniality today (Ciocchini & Greener, 2021).

In many ways, this article poses more questions than it answers, illustrative of its purpose. How can we develop better, more expansive global research collaborations, challenge coloniality and recentre authenticity and connection in methodologies? How can we engage in a sensitive, critical, and contemplative process of the impact of colonialism on research and researchers? How can we affect ‘real’ change, and use research to better protect vulnerable children and halt the spread of organised crime?

## Footnotes

1 Discussed in a number of other publications, including Atkinson-Sheppard, 2019; 2017a; 2017b).

2 King’s College London ethical committee and the University of Westminster, British Psychological Society codes of ethical conduct.

3 Not his real name.

4 A term used to describe an organised crime boss/group.

5 Not his real name.

6 Unstructured interview 17 (Bangladesh).

7 All of which are discussed in associated publications.

## References

[CR1] Agozino B (2003). Counter-colonial criminology: A critique of imperialist reason.

[CR2] Atkinson-Sheppard S (2019). *The gangs of Bangladesh*; *Mastaans, street gangs and ‘illicit child labourers’ in Dhaka*.

[CR3] Atkinson-Sheppard S, Hayward H (2019). Conceptual similarities; distinct difference: Exploring ‘the gang’ in Mainland China. British Journal of Criminology..

[CR4] Atkinson-Sheppard S (2017). Street children and Dhaka’s gangs: Using a case study to explore Bangladeshi organised crime. SAGE Research Methods Cases.

[CR5] Atkinson-Sheppard S (2017). Street children and ‘protective agency’: Exploring young people’s involvement in organised crime in Dhaka, Bangladesh. Childhood.

[CR6] Bakken B (2018). Crime and the Chinese dream.

[CR7] Blanchet T (2008). Lost innocence, stolen childhoods.

[CR8] Blagg H, Anthony T (2019). Decolonising criminology: Imagining justice in a post-colonial world.

[CR9] Bowling B (2010). Policing the Caribbean: Transnational security cooperation in practice.

[CR10] Bryman A, Cassell C (2006). The researcher interview: A reflexive perspective. Qualitative Research in Organisations and Management..

[CR11] Carrington, K., Hogg, R., Scott, J., & Sozzo, M. (2018) Criminology, southern theory and cognitive justice in Carrington et al (eds) *The palgrave handbook of criminology and the global south*. Switzerland: Palgrave Macmillain.

[CR12] Carrington K, Hogg R, Sozzo M (2016). Southern criminology. British Journal of Criminology.

[CR13] Chan, M., K., (1996) Colonial legacy, transformation and challenge. *The ANNALS of the American Academy of Political and Social Science. Vol. 547:1* page(s): 11–23*.*

[CR14] Connell R (2007). Southern theory: The global dynamics of knowledge in the social sciences.

[CR15] Ciocchini, P. and Greener, J. (2021) Mapping the pains of neo-colonialism: A critical elaboration of southern criminology. *The British Journal of Criminology*, 2021, XX, 1–18.

[CR16] Cunneen C (2011). Indigeneity, sovereignty and the law: Challenging the processes of criminalisation. South Atlantic Quarterly.

[CR17] Cunneen C, Rowe S (2014). Changing narratives: Colonised peoples, criminology and social work. International Journal for Crime, Justice and Social Democracy..

[CR18] Dimou E (2021). Decolonising southern criminology: What can the ‘decolonial option’ tell us about challenging the modern/colonial foundations of criminology?. Critical Criminology.

[CR19] Englander M (2012). The interview: Data collection in descriptive phenomenological human scientific research. Journal of Phenomenological Psychology.

[CR20] Gurung, T., I. (2018) Why the British never colonised Nepal. Available online: https://asiatimes.com/2018/05/why-the-british-never-colonized-nepal/ [Accessed: 16^th^ June 2021].

[CR21] Fun, Y. and Yao, Z. (2018) A state of contradiction: Medical corruption and strain in Beijing public hospitals Bakken, B. eds. (2018) *Crime and the Chinese Dream*. Hong Kong: Hong Kong University Press.

[CR22] Jaishankar K (2020). Routledge handbook of South Asian criminology.

[CR23] Keane, M., Khupe, C. and Seehawer, M. (2017) Decolonising methodology: Who benefits from indigenous knowledge research? Educational Research for Social Change (ERSC) 6, 1, April 2017, 12–24

[CR24] Khatun MT, Jamil H (2013). Lifestyle of the street children in Khulna City. Bangladesh Research Publications Journal..

[CR25] Khondaker, M., I.; Kashem, M., B. and Rahman, M., A. (2020) Bangladesh: Issues and introspection on crime and criminal justice in Jaishankar, K. eds. (2020) *Routledge Handbook of South Asian Criminology*. New York: Routledge.

[CR26] Lewis D (2012). Bangladesh: Politics, economy and civil society.

[CR27] Liebel M (2020). Decolonizing childhoods.

[CR28] Liu (2017). Asian paradigm theory and access to justice. Journal of Contemporary Criminal Justice.

[CR29] Mahmud, F. (2019) Adda: what the institution of the convivial community chat over ‘cha’ means for Bengali literature. Available online at: https://scroll.in/article/940880/adda-what-the-institution-of-the-convivial-community-chat-over-cha-means-for-bengali-literature [Accessed 20 March 2021].

[CR30] Maldonado-Torres N (2007). On the coloniality of being. Cultural Studies.

[CR31] Mignolo, W. D. (1995) Decires fuera du lungar; sujetos dicentes, roles sociales y formas de inscription ‘ *Revista de critica literaria LationAmericano* Vol. 11: 9–32.

[CR32] Mignolo WD, Walsh C (2019). On decoloniality.

[CR33] Moosavi L (2019). (2019) A friendly critique of ‘Asian Criminology’ and ‘Southern criminology’. British Journal of Criminology..

[CR34] Moniruzzaman M (2009). Party politics and political violence in Bangladesh: Issues, manifestation and consequences. South Asian Survey..

[CR35] Morrison H (2012). The global childhood of history reader.

[CR36] Nipun, U., Q. (2020) Transitional justice process in Bangladesh in Jaishankar, K. eds. (2010) *Routledge Handbook of South Asian Criminology*. New York: Routledge.

[CR37] Said E, W.  (1978). Orientalism.

[CR38] Sharma, M. (2020) Nepal: Institutions in the criminal justice system and the need for reforms in Jaishankar, K. eds. (2010) *Routledge Handbook of South Asian Criminology*. New York: Routledge.

[CR39] Silverman, D. (2010) *Qualitative research* (3^rd^ Edt.) London: Sage.

[CR40] Sitoula, S., B. (2020) Human trafficking in Nepal: A victimology perspective in Jaishankar, K. eds. (2010) *Routledge Handbook of South Asian Criminology*. New York: Routledge.

[CR41] Subedi, D., B. (2020) Post-conflict crime and violence in Nepal: Trends, dynamics and drivers in Jaishankar, K. eds. (2010) *Routledge Handbook of South Asian Criminology*. New York: Routledge.

[CR42] Temple B (1997). Watch your tongue: Issues in translation and cross-cultural research. Sociology.

[CR43] Temple B (2002). Crossed wires: Interpreters, translators, and bilingual workers in cross-language research. Qualitative Health Research..

[CR44] Thapa, R. R. (2019) State fragility and organized crime. Available online: https://www.nepjol.info/index.php/JAPFCSC/article/download/26752/22144/ [Accessed 26 April 2021].

[CR45] Uddin, M.K (2018) A southern perspective on extrajudicial killings in Bangladesh in Carrington, K., Hogg, R., Scott, J. and Sozzo, M. (eds.) *Palgrave Handbook of Southern Criminology.* London: Palgrave.

[CR46] UNICEF (2012) Child rights Bangladesh: UNICEF [Online]. Available at: http://www.unicef.org/bangladesh/children_4878.htm [Accessed 25 January 2021].

[CR47] Vannini P, Fanzese A (2008). The authenticity of self: Conceptualization, personal experience and practice. Sociology Compass.

[CR48] Wang P (2017). The Chinese mafia: Organized crime, corruption, and extra-legal protection.

[CR49] Wong  KC (2002). Policing in the People’s Republic of China: The road to reform in the 1990s. British Journal of Criminology..

[CR50] Wong R (2019). The illegal wildlife trade in China: Understanding the distribution networks.

[CR51] Xu J (2009). The robbery of motorcycle taxi drivers in China: A lifestyle/routine activity perspective and beyond. British Journal of Criminology..

[CR52] Xu J, Laider K, J. and Lee, M.  (2013). Doing criminological ethnography in China: Opportunities and challenges. Theoretical Criminology..

[CR53] Yin, R. K. (2014). *Case study research: Design and methods* (5th ed.). Sage.

[CR54] Zavala, M. (2013) What do we mean by decolonizing research strategies? Lessons from decolonizing, indigenous research projects in New Zealand and Latin America, decolonization: *Indigeneity, education & society*, 2, 1, 55-71.

[CR55] Zhang L, Messner S, F., Liu, J., Zhuo, Y. A.  (2009). Guanxi and fear of crime in contemporary urban China. The British Journal of Criminology..

